# Multiple Intraosseous Cysts of the Carpal Bones Presenting as Unilateral Carpal Tunnel Syndrome

**DOI:** 10.1155/2023/4110616

**Published:** 2023-05-24

**Authors:** Raymonde Dahdouh, Dany Aouad, Elyssa Kiwan, Georges Sakhat, Mohammad Daher, Rabih Kortbawi, Joseph Wehbe

**Affiliations:** ^1^Department of Orthopedic Surgery and Traumatology, Saint George Hospital University Medical Center, Balamand University, P.O. Box 166378, Achrafieh, Beirut 1100 2807, Lebanon; ^2^Faculty of Medicine, Saint-Joseph University, P.O. Box 17-5208, Mar Mikhael, Beirut, Lebanon

## Abstract

Intraosseous ganglion cysts (IGC) of the carpal bones are frequently reported in the literature, involving at most two carpal bones of the same wrist. Only one case recently described the presence of multiple intraosseous ganglion lesions in the capitate, lunate, and triquetrum, resulting in chronic wrist pain. The following study reports the first case of multiple IGCs causing a unilateral carpal tunnel syndrome (CTS), in a 56-year-old woman, with no previous history of trauma. Failure of conservative management prompted carpal tunnel release and the surgical excision of the ICGs, followed by autologous bone grafting to fill in the defects. Consequently, IGCs must be considered in the differential diagnosis of unilateral CTS due to the expansile nature of the bone lesions.

## 1. Introduction

Intraosseous ganglion cysts (IGC) of the carpal bones have long been reported in literature [1]. They are benign, non-neoplastic, and mucin-filled lesions [2] that develop within a bone, with or without communication with an adjacent joint [3]. Although they have been described as the most frequent bone lesions occurring in the carpus [4], mainly in the scaphoid or the lunate [5], only one case has reported the simultaneous presence of cysts in more than two carpal bones of the same wrist to date [6]. Moreover, most carpal IGC are asymptomatic, and thus, incidentally discovered on plain radiographs [5]. Less frequently, they are associated with chronic wrist pain, irrespective of a history of wrist trauma [7]. Darcy et al. were the first and only ones to describe a strictly IGC in the capitate as a cause of unilateral carpal tunnel syndrome (CTS) [3]. Usually, it is common to think of a space-occupying lesion in the carpal tunnel, such as a mass or a cyst, as an underlying etiology for median nerve compression [8]; however, the expansile nature of the intraosseous cyst makes its diagnosis worthy to consider in the differential of a unilateral CTS [3]. We document the first case of unilateral CTS secondary to the simultaneous existence of IGC in three different carpal bones of the same wrist.

## 2. Case Presentation

A 56-year-old woman, with a history of hypertension, presented to the clinic for non-resolving atraumatic left wrist pain. Several months prior to presentation, the patient started experiencing pain in her left wrist. She resorted to the usage of analgesics and splinting, which provided only partial relief of pain. Upon presentation, examination revealed no visible erythema, swelling, or deformity, but was significant for positive Tinel (tingling or shock-like sensation felt when tapping over the median nerve) and Phalen (elicits numbness and tingling in the hand and fingers by holding the wrist in a flexed position) signs, with reproducible pain and paresthesia over the wrist and the first three and a half fingers. Motor function of the median, radial, and ulnar nerves was preserved.

**Figure 1:**
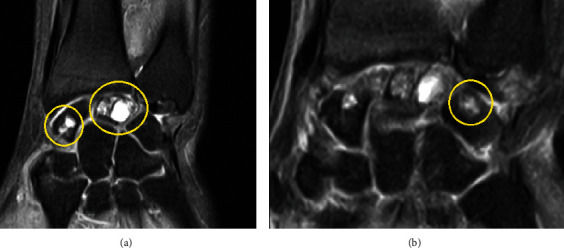
MRI of the left wrist showing IGC (from (a) to (b)) in the scaphoid, lunate (a), and triquetrum (b).

The patient underwent an electromyography (EMG), which showed median nerve compression at the level of the wrist consistent of CTS. A magnetic resonance imaging (MRI) of the left wrist was done due to the atypical presentation of her wrist pain, revealing three benign appearing cystic lesions attributable to IGC. A 7 mm × 8 mm × 9 mm cystic lesion was found in the lunate bone of decreased and increased signal on T1 and T2 weighted images, respectively, associated with trabecular thickening and enlargement of the bone with no cortical erosion. A 6 mm × 7 mm × 5 mm multi-septate intra-medullary lesion was found in the distal two-thirds of the scaphoid bone, and a similar smaller lesion was noted in the proximal and medial aspect of the triquetrum; both of which were surrounded by a regular sclerotic rim without adjacent bone marrow edema or erosion of the cortex (Figure 1).

**Figure 2:**
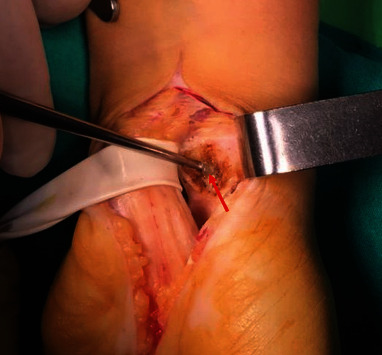
IGC (red arrow) of the lunate bone with the carpal tunnel contents retracted using a Penrose.

The patient was scheduled for surgical excision of the ganglion cysts and carpal tunnel release of the left wrist. Under left upper limb nerve block, a curved incision ulnar to and paralleling the thenar crease was done with extension to the proximal aspect of the palm. Identification of the deep fascia of the forearm proximal to the carpal tunnel by subcutaneous blunt dissection, and incision of the fascia avoiding injury to the median nerve beneath it. Identification of the distal end of the transverse carpal ligament, and careful division of the transverse carpal ligament along its ulnar border to avoid damage to the median nerve and release of all components of the flexor retinaculum. Using a Penrose (Figure 2), retraction of the carpal tunnel contents medially and laterally to expose triquetrum, scaphoid, and lunate bones, respectively.

Using a burr, a bony window was done in each of the bones under fluoroscopic guidance. Then, curettage and excision of the intraosseous bone cysts were done. Later on, proximal extension of the incision down to distal radius was performed followed by creation of a small cortical window and removal of bone graft, which was then inserted in the curetted carpal bones. This was followed by repair of the carpal ligaments, and closure was done by layers over a medium sized drain, after which an extension wrist brace was put in place.

At 1, 3, and 6 months follow up, patient had resolution of all her previously reported symptoms with return to her previous activities.

## 3. Discussion

CTS is usually idiopathic, but secondary causes exist and include inflammatory conditions, vascular abnormalities, malunited fractures, and space-occupying lesions [8]. It has previously been uncommon to use imaging modalities for the investigation and diagnosis of bilateral CTS. However, it is important to consider wrist imaging in unilateral CTS to rule out space-occupying lesions, which have an incidence of 5.5% [3] especially if the patient had an atypical presentation such as our patient. The differential diagnosis in such cases with unilateral CTS and progressive wrist pain without underlying trauma includes ganglion cysts, giant cell tumor, chondroblastoma, osteoid osteoma, osteoblastoma, and osteoarthritis [3].

Intraosseous carpal cysts (ICC) are frequently asymptomatic, found incidentally on wrist radiographs as lytic lesions while investigating other conditions of wrist pain, and are most commonly found in the proximal row of carpal ones [5] such as our patient where the ICC were found in the scaphoid, lunate, and triquetrum. The etiology and pathophysiology of ICC remain uncertain, with two proposed mechanisms. It is reported that soft tissue ganglia penetrate the cortices of carpal bones, thus called the penetration theory, and studies have shown that 47% of extraosseous ganglia are associated with IGC. The idiopathic theory, proposed by multiple authors, suggest that microtrauma and repetitive stress to carpal bones lead to metaplastic change of mesenchymal stem cells into synovial cells as a result of intramedullary vascular disturbance; this explains the higher incidence of ICC in proximal carpal bones specifically the lunate and scaphoid [5, 7]. Several cases of ICC involving one or two carpal bones have been reported, whereas cases where multiple carpal bones are simultaneously involved remain very rare.

In one case report written by Ali et al., a 56-year-old female was found to have IGC in the lunate, capitate, and triquetrum after undergoing an MRI for wrist pain. The patient in the aforementioned case report opted for conservative management with oral analgesics and splinting rather than operative treatment [6]. Furthermore, a case report by Darcy et al. reported a CTS caused by an intraosseous ganglion of the capitate; however, operative management was conducted [3]. A case where intraosseous cysts were compressing the ulnar nerve was as well reported in the literature [9]. Conservative management is preferred for asymptomatic lesions and some symptomatic cases, with observation and monitoring, anti-inflammatory medications or infiltrations, and lifestyle modifications. In cases of rapid progression, cortical erosion, pathologic fractures, or non-responsiveness to conservative measures, surgical intervention is indicated. Our patient did not respond to conservative management making surgery the most reasonable approach. According to literature, the optimal surgical approach is curettage of the lesion along with extensive irrigation, followed by autologous bone grafting from the radius or iliac crest to prevent fracture [5]. A study by Yu et al. suggest the use of bone cement injection as an alternative to autogenous bone grafting. Although the study suggests that the method is effective and safe, one limitation is the risk of bone cement leakage into the wrist joint in case of disruption of the cortex [2].

## 4. Conclusion

IGC, involving multiple carpal bones simultaneously, and presenting as CTS, are a very rare entity. Its diagnosis is highly dependent on imaging studies that investigate unilateral, rather than bilateral, median nerve compressions. Surgical management is preferred once conservative approaches fail, and bone grafting is encouraged to avoid consequent fractures.

## Data Availability

Data supporting this research article are available from the corresponding author or first author on reasonable request.

## References

[1] Magee T. H., Rowedder A. M., Degnan G. G. (1995). Intraosseous ganglia of the wrist. *Radiology*.

[2] Yu K., Shao X., Tian D., Bai J., Zhang B., Zhang Y. (2016). Therapeutic effect of bone cement injection in the treatment of intraosseous ganglion of the carpal bones. *Experimental and Therapeutic Medicine*.

[3] Darcy P. F., Sorelli P. G., Qureshi F., Orakwe S., Ogufere W. (2004). Carpal tunnel syndrome caused by an intraosseous ganglion of the capitate. *Scandinavian Journal of Plastic and Reconstructive Surgery and Hand Surgery*.

[4] Van den Dungen S., Marchesi S., Ezzedine R., Bindou D., Lorea P. (2005). Relationship between dorsal ganglion cysts of the wrist and intraosseous ganglion cysts of the carpal bones. *Acta Orthopaedica Belgica*.

[5] Osagie L., Gallivan S., Wickham N., Umarji S. (2015). Intraosseous ganglion cysts of the carpus: current practice. *Hand*.

[6] Ali M., Walker T., Mannan S. (2018). Simultaneous triquetrum, lunate, and capitate interosseous ganglion cysts. *Journal of Orthopaedic Case Reports*.

[7] Ikeda M., Oka Y. (2000). Cystic lesion in carpal bone. *Hand Surgery*.

[8] Yalcinkaya M., Akman Y. E., Bagatur A. E. (2014). Unilateral carpal tunnel syndrome caused by an occult ganglion in the carpal tunnel: a report of two cases. *Case Reports in Orthopedics*.

[9] Al-Qattan M. M. (2004). A penetrating intraosseous ganglion of the triquetrum causing ulnar nerve compression. *Hand Surgery*.

